# An alternative to hydrogenation processes. Electrocatalytic hydrogenation of benzophenone

**DOI:** 10.3762/bjoc.14.40

**Published:** 2018-03-01

**Authors:** Cristina Mozo Mulero, Alfonso Sáez, Jesús Iniesta, Vicente Montiel

**Affiliations:** 1 Instituto de Electroquímica, Universidad de Alicante, Apartado 99, 03080 Alicante, Spain

**Keywords:** benzophenone, diphenylmethanol, electrocatalytic hydrogenation, palladium nanoparticles, polymer electrolyte membrane

## Abstract

The electrocatalytic hydrogenation of benzophenone was performed at room temperature and atmospheric pressure using a polymer electrolyte membrane electrochemical reactor (PEMER). Palladium (Pd) nanoparticles were synthesised and supported on a carbonaceous matrix (Pd/C) with a 28 wt % of Pd with respect to carbon material. Pd/C was characterised by transmission electron microscopy (TEM), and thermogravimetric analysis (TGA). Cathodes were prepared using Pd electrocatalytic loadings (L_Pd_) of 0.2 and 0.02 mg cm^−2^. The anode consisted of hydrogen gas diffusion for the electrooxidation of hydrogen gas, and a 117 Nafion exchange membrane acted as a cationic polymer electrolyte membrane. Benzophenone solution was electrochemically hydrogenated in EtOH/water (90/10 v/v) plus 0.1 M H_2_SO_4_. Current densities of 10, 15 and 20 mA cm^−2^ were analysed for the preparative electrochemical hydrogenation of benzophenone and such results led to the highest fractional conversion (X_R_) of around 30% and a selectivity over 90% for the synthesis of diphenylmethanol upon the lowest current density. With regards to an increase by ten times the Pd electrocatalytic loading the electrocatalytic hydrogenation led neither to an increase in fractional conversion nor to a change in selectivity.

## Introduction

Hydrogenation is a common procedure applied in organic chemistry industry based on the use of an external hydrogen source, generally carried out under moderate experimental conditions of high temperature (until 673 K) and high-pressure (even 350 atm). Even though the hydrogenation is performed using either homogeneous or heterogeneous catalysts to enhance chemical kinetics, a number of side reactions reduces the selectivity of the chemical reaction providing also complex and cost-ineffective work-up procedures [1–3]. Alternatively, electrocatalytic hydrogenation has emerged as a technique driven by its operational mild conditions, i.e., in situ “active hydrogen” generation, room temperature and atmospheric pressure, and higher selectivity [4–10]. This electrochemical “active hydrogen” generation is performed under a cathodic polarization and such mechanism of an unsaturated organic compound (e.g., Y=X) still remains undertrained. Nonetheless, the steps likely involved in this electrochemical process by the literature [11–15] are as follows: (i) formation of adsorbed hydrogen MH_ads_ (defining “M” as a metal adsorption site and H_ads_ as atomic adsorbed hydrogen), (ii) adsorption of the organic molecule on a support site (two distinct adsorption sites are considered) and, (iii) electrocatalytic hydrogenation of the organic molecule through the adsorbed atomic hydrogen. It is important to note that, formation of “active hydrogen” is the main step in this process and hydrogen-active powder electrocatalysts such as Pd/C, Pt/C or Raney-nickel have been demonstrated as the optimal choice [16–17]. Moreover, the organic molecule adsorption rate must be faster than that one associated with the recombination of MH_ads_ to form diatomic hydrogen gas; otherwise electrocatalytic hydrogenation does not occur or displays a low Faraday efficiency because of the fact that diatomic hydrogen formation is not the main reaction, but a competitive one. Clearly, from the above mechanistic steps, the lower the “atomic active hydrogen” generation rate, the more efficient the electrocatalytic hydrogenation process; thus, the higher the used current density, the lower the obtained efficiency. Moreover, selectivity of electrocatalytic hydrogenation of ketones to alcohols is performed in acidic medium as an optimal reaction medium [5].

Currently, developments in nanostructured materials and polymeric solid exchange membranes have led the field of polymer electrolyte membrane fuel cells (PEMFC) to become a mature technology [18–20]. In this regard, a polymer electrolyte membrane electrochemical reactor (PEMER) has already been defined and manufactured to implementing all PEMFC technology advantages for both inorganic and organic electrosynthetic processes [21]. It is worth noting that the use of a polymer electrolyte membrane electrochemical reactor (PEMER) allows obtaining several advantages compared to the use of conventional electrochemical reactors, as follows: i) nanostructurated electrocatalysts can be utilized for both cathodic and anodic reactions, ii) a solid polymer electrolyte is used instead of a conventional electrolyte and iii) a decrease of the anode–cathode gap reduces ohmic drop at the whole process. In the case of organic electrosynthesis, electrocatalytic hydrogenation of aromatic ketones, specifically acetophenone, has been recently carried out [22–23] achieving a high selectivity using the above-mentioned technology. In this work, we have chosen benzophenone, as a more complex aromatic ketone dictated by the presence of two phenyl rings, in order to analyse fractional conversion and selectivity of formed products. Here, the main obtained product is diphenylmethanol (ketone conversion to alcohol) as a high added value product in chemical industry. Even though several research groups have reported in the literature fundamental aspects about the electrocatalytic hydrogenation of benzophenone as well as its electrolytic performance at laboratory scale using Pd, Ni/Pd or Ni/Pt based either on massive or nanoparticulate cathodes [5,24–26]. The electrocatalytic hydrogenation of benzophenone has not been accomplished using a PEMER yet.

This work aims at exploring the electrocatalytic hydrogenation of benzophenone over palladium nanoparticles supported on carbon black based electrode using a PEMER. A nanoparticulate platinum gas diffusion electrode is used for the hydrogen oxidation reaction and a cationic exchange membrane is used as solid polymeric electrolyte. Fractional conversion, product yield and selectivity are presented upon the Pd electrocatalytic loading and current density. Moreover, the effect of the absence and presence of the supporting electrolyte upon the conversion and product yield of the electrochemical hydrogenation of benzophenone is explored.

## Results and Discussion

**Figure 1:**
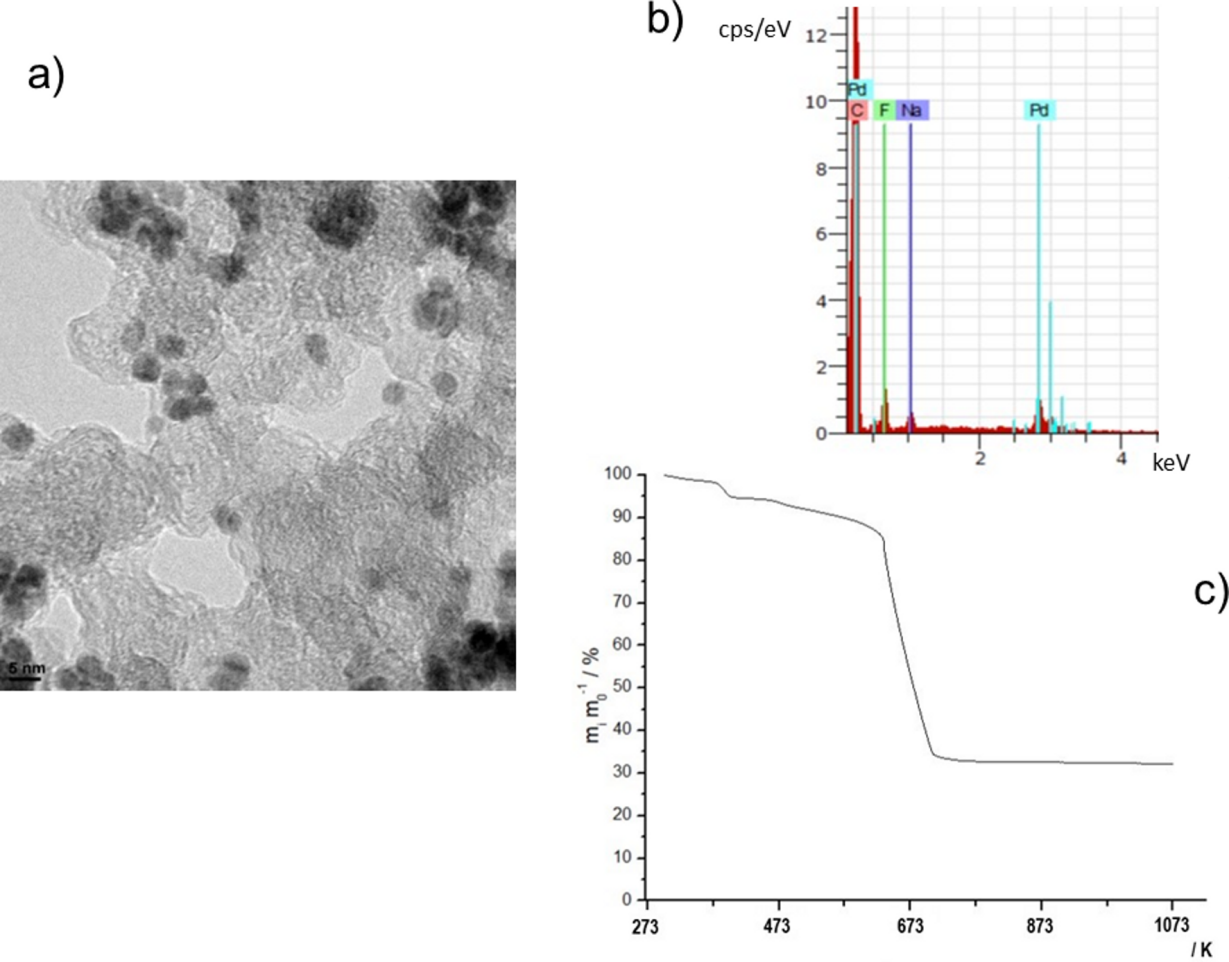
Characterisation of Pd/C electrocatalyst. a) TEM micrograph. b) Energy dispersive X-ray analysis (EDX). c) Thermogravimety analysis in N_2_/O_2_ (4/1) atmosphere at a heating slope of 10 K min^−1^ from 298 K to 1023 K.

Pd nanoparticles supported on Vulcan XC72R as electrocatalyst in cathodic reaction (electrocatalytic hydrogenation) were synthesised by reducing K_2_PdCl_4_ using NaBH_4_ in a water-in-oil (w/o) microemulsion (water/Brij@30/*n*-heptane). This methodology has been previously used in our laboratory [27–28]. We first explored the morphology, size and dispersion of Pd nanoparticles supported on Vulcan XC72R carbonaceous material (Pd/C electrocatalysts) using TEM micrographs. As shown in Figure 1a, a good dispersion of spherical Pd nanoparticles of about 4–5 nm size is obtained. As expected, EDX analysis of Pd nanoparticles displays palladium as the sole metal, as shown in Figure 1b. With respect to the Pd loading in the Pd/C electrocatalyst, TGA results shown in Figure 1c lead to ca. 30 wt % content. A high drop of weight is observed between 600 K and 700 K since carbonaceous support is volatilized until reaching a residual PdO content (Pd in a N_2_/O_2_ atmosphere becomes a PdO species as stable residual weight when temperature is increased from 700 K to 1073 K). From the stable residual weight in Figure 1c at higher temperature it can be estimated that there is a content of 32 wt % of PdO in the whole Pd/C electrocatalyst, thereby confirming that the electrocatalyst can be considered as Pd/C with 28 wt %, by correcting the PdO value by subtracting the oxygen atomic percentage content. Thus, the above experimental wt % value of Pd is in agreement with the theoretical one of 30 wt % as described in the experimental section.

**Figure 2:**
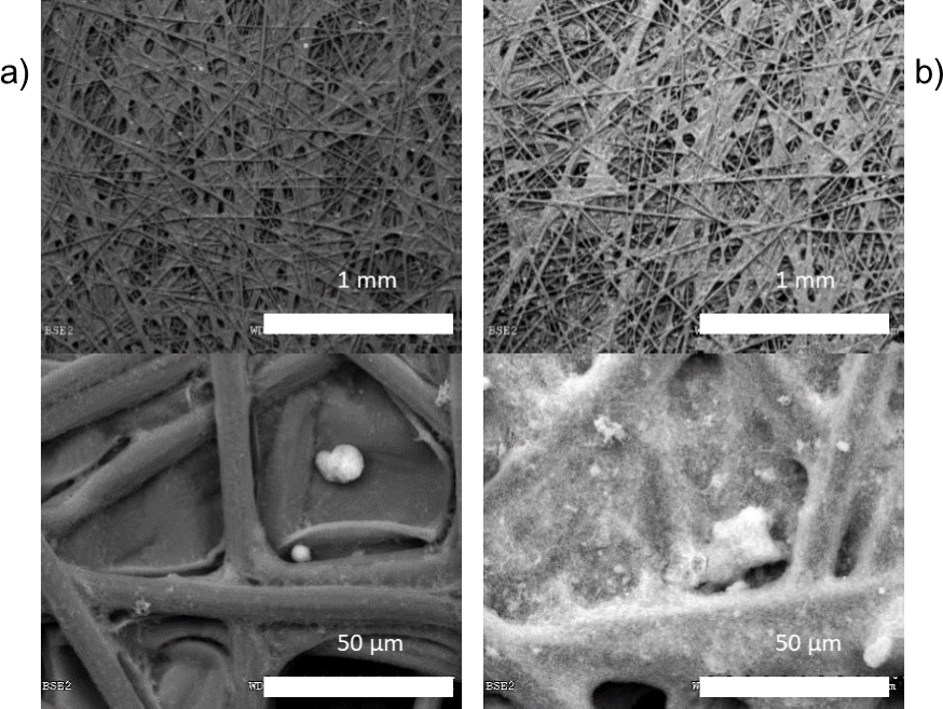
SEM images of (a) Pd_0.02_/C/T and (b) Pd_0.20_/C/T electrodes, with different magnifications.

Once the Pd/C electrocatalyst characterisation was performed, Pd/C based cathodes either with 0.2 or 0.02 mg Pd per cm^2^ were prepared and characterised by SEM and cyclic voltammetry. A Pd/C to Nafion ratio was established at 60:40 for each electrocatalytic layer irrespectively of the Pd loading in the electrocatalytic layer. Figure 2 depicts the SEM micrographics using back scattering electrons for both Pd_0.02_/C/T and Pd_0.20_/C/T electrodes (nomenclature described in the experimental section) at different magnifications. The SEM image for the highest electrocatalytic loading discloses a brighter appearance compared to the lowest one since backscattering electrons from high atomic weight (e.g., palladium) are brighter than lower atomic weight (e.g., carbon).

**Figure 3:**
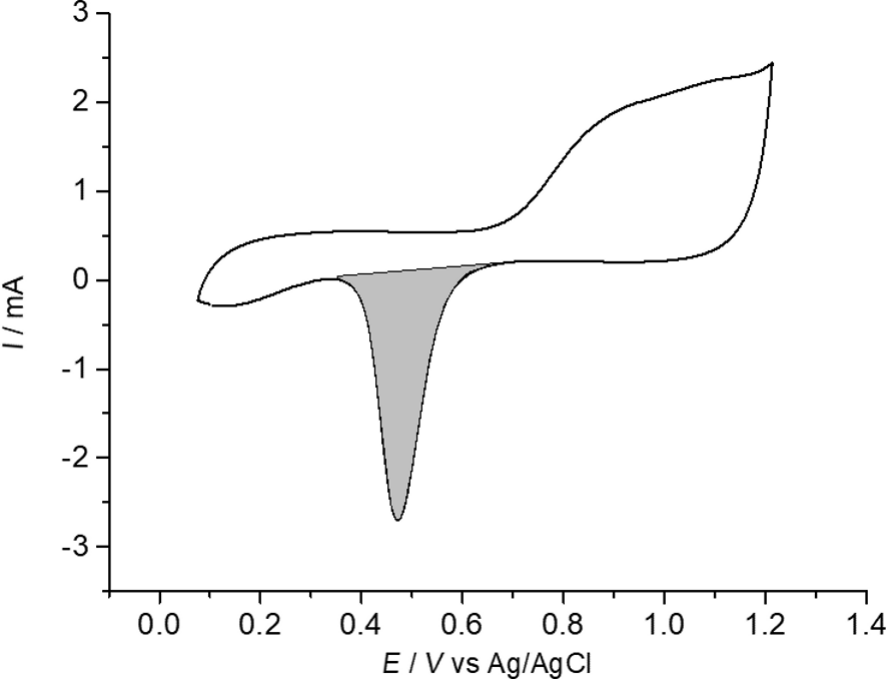
Cyclic voltammetric behaviour of Pd_0.20_/C/T electrode in 0.5 M H_2_SO_4_. Scan rate: 50 mV s^−1^. Starting potential: 0.2 V vs Ag/AgCl. Positive scan.

Apart from the SEM characterisation, the determination of the electrochemically active surface area (ESA) of the cathodic electrocatalytic layer is also crucial for the characterisation of both electrodes. In this regard, Figure 3 depicts the electrochemical behaviour of a Pd_0.20_/C/T electrode in 0.5 M H_2_SO_4_ using cyclic voltammetry (CV) at a scan rate of 50 mV s^−1^ (potential interval of 0.1 and 1.2 V vs Ag/AgCl). The CV diagram shown in Figure 3 represents a typical pattern for the electrochemical behaviour of Pd bar electrode where a broad anodic peak is associated to the oxidation of the Pd surface to PdO and then followed by a well-defined cathodic peak on the negative scan ascribed to the reduction of PdO to Pd [29]. From the coulombic charge integration of the cathodic peak on the CV in Figure 3 (shadowed region) can be accurately calculated the ESA value and roughness factor, as defined in the experimental section. The ESA value is calculated according to Equation 1,

[1]
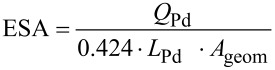


where *Q*_Pd_ (mC) is calculated from the coulombic charge integration from the cathodic peak after subtracting the double layer, *L*_Pd_ denotes the electrocatalytic loading expressed in mg of Pd per square centimetre of electrocatalytic layer, *A*_geom_ (cm^2^) is the electrode geometric area and 0.424 refers to charge density expressed in mC per cm^2^ of Pd [29]. On the other hand, the roughness factor rf is also calculated according to Equation 2.

[2]
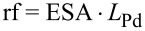


Calculations of ESA and rf values were addressed for both Pd_0.02_/C/T and Pd_0.20_/C/T electrodes. It is worth noting that rf values denote real electrocatalytic surface area as a multiple factor of the theoretical geometric area. In this regard, rf values were 85 and 14 times the geometric area, whereas ESA values were 707 and 424 for Pd_0.02_/C/T and Pd_0.20_/C/T electrodes, respectively. The later ESA values indicate an increase of 67% of active surface area per weight unit, so the higher the electrocatalytic loading shown in a cathode electrocatalytic layer, the lower the electrochemically accessible surface per milligram. The above behaviour is likely attributed to a much thicker electrocatalytic layer for the Pd_0.20_/C/T electrode compared to Pd_0.02_/C/T electrode. Nonetheless, a thicker electrocatalytic layer is also detrimental for the accessibility of the electroactive species to the electrocatalytic metal surface, i.e., Pd nanoparticles, mostly due to the appearance of bottlenecks or blockage of network across the electrocatalytic layer, or likely some of these Pd nanoparticles could be covered up each other so those ones would be hidden.

**Figure 4:**
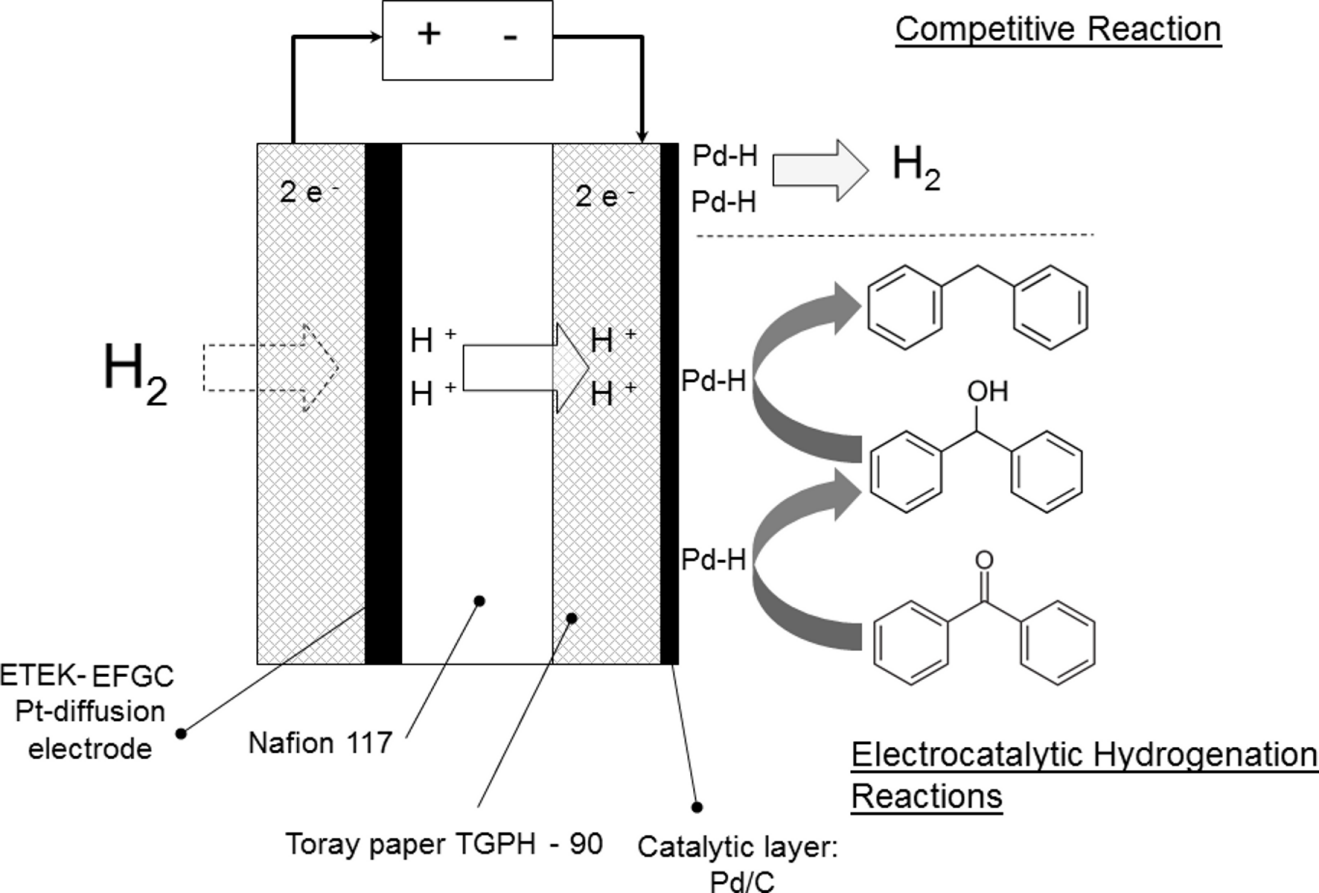
Scheme of all components of the electrochemical reactor including reactions involved in both anode and cathode.

Once Pd nanoparticle-based cathodes were manufactured and characterised, electrolytic hydrogenation of benzophenone in a cathodic process and hydrogen oxidation reaction as the anodic process was performed using a PEMER. Figure 4 describes each one of reactions involved in both cathodic and anodic processes and, also, the particulate elements of the electrochemical reactor.

**Figure 5:**
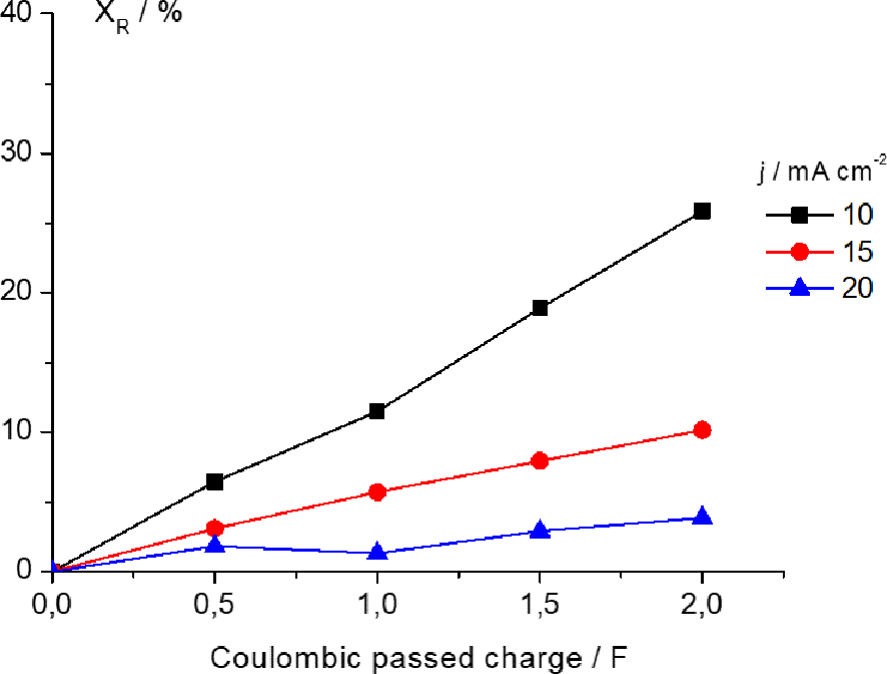
Fractional conversion of benzophenone as a function of coulombic passed charge using the PEMER; 0.5 M benzophenone as starting solution in ethanol/H_2_O (90:10 v/v) + 0.1 M H_2_SO_4_. Pd_0.02_/C/T electrode.

Thus, preparative electrocatalytic hydrogenation of benzophenone was performed using a PEMER with Pd_0.02_/C/T and Pd_0.20_/C/T electrodes. We first investigated the influence of current density, i.e., 10, 15 and 20 mA cm^−2^, on the electrochemical hydrogenation using a Pd_0.02_/C/T electrode with a coulombic charge passed of 2 F (theoretical coulombic charge established by Faraday’ s Law by considering reaction of one mol of reagent). Figure 5 depicts the fractional conversion of benzophenone (expressed in percentage) versus the coulombic charge passed for all three current densities examined.

It should be noted that a higher fractional conversion of benzophenone of 25.8% is obtained for the lowest current density compared with values of 10.1% and 3.9% for current densities of 15 and 20 mA cm^−2^, respectively (see Table 1). Our findings are highly expected accordingly to the steps involved in the electrochemical hydrogenation mechanisms (vide supra). In this regard, after formation of PdH_ad_, the recombination rate of this “atomic active hydrogen” into diatomic hydrogen gas should be slower than the adsorption rate of the organic compound over the cathode in order to prompt electrocatalytic hydrogenation; consequently, a low current density should favour the hydrogenation process instead of diatomic hydrogen formation as competitive and side reaction by considering a slow PdH_ad_ formation rate of this.

**Table 1:** Fractional conversion (X_R_), product yield for diphenylmethanol (η_Ph2CHOH_) and diphenylmethane (η_Ph2CH2_), selectivity for diphenylmethanol (ξ_Ph2CH2_) versus coulombic charge passed at constant current densities of 10, 15 and 20 mA cm^−2^. Pd_0.02_/C/T electrode. Benzophenone (0.5 M) was used as starting solution in ethanol/water (90:10 v/v) + 0.1M H_2_SO_4_.

*j*			*Q*_passed_	[F]		∆*U*_cell_
		
[mA cm^−2^]		0.5	1.0	1.5	2.0	[V]

	X_R_	6.5	11.5	18.9	25.8	
10	η _Ph2CHOH_	4.1	9.2	16.9	22.4	0.2
	η _Ph2CH2_	0.9	1.1	1.6	1.6	
	ξ _Ph2CHOH_	83	89	91	93	

	X_R_	3.1	5.7	7.9	10.1	
15	η _Ph2CHOH_	3.7	5.4	7.4	9.9	0.3
	η _Ph2CH2_	0	0	0.3	0.4	
	ξ _Ph2CHOH_	100	100	96	96	

	X_R_	1.8	1.3	2.9	3.9	
20	η _Ph2CHOH_	0.9	1.7	2.5	3.0	0.5
	η _Ph2CH2_	0	0	0.3	0.4	
	ξ _Ph2CHOH_	100	100	88	88	

HPLC analysis was performed for the identification and quantification of the benzophenone depletion and final product formation from the electrocatalytic hydrogenation at Pd_0.02_/C/T electrode. An inspection of the chromatograms confirms the formation of two final products corresponding to diphenylmethanol (Ph_2_CHOH) and diphenylmethane (Ph_2_CH_2_). Table 1 compiles fractional conversion of Ph_2_CO (X_R_), product yield of Ph_2_CHOH and Ph_2_CH_2_ (η), selectivity of Ph_2_CHOH (ξ) and cell voltage (∆*U*_cell_) for each current density over coulombic charge passed. It is worth noting that product yield for the Ph_2_CHOH is higher at low current density with an almost linear increase as a function of coulombic charge passed. Moreover, it is noticed that the selectivity value generally over 90% is irrespective of the current density. Electrocatalytic hydrogenation of benzophenone was also performed using Pd_0.00_/C/T electrodes, i.e., in the absence of the Pd electocatalyst, at 10 mA cm^−2^ demonstrated neither the electrochemical reduction of benzophenone nor the formation of alcohol or alkane derivatives.

**Figure 6:**
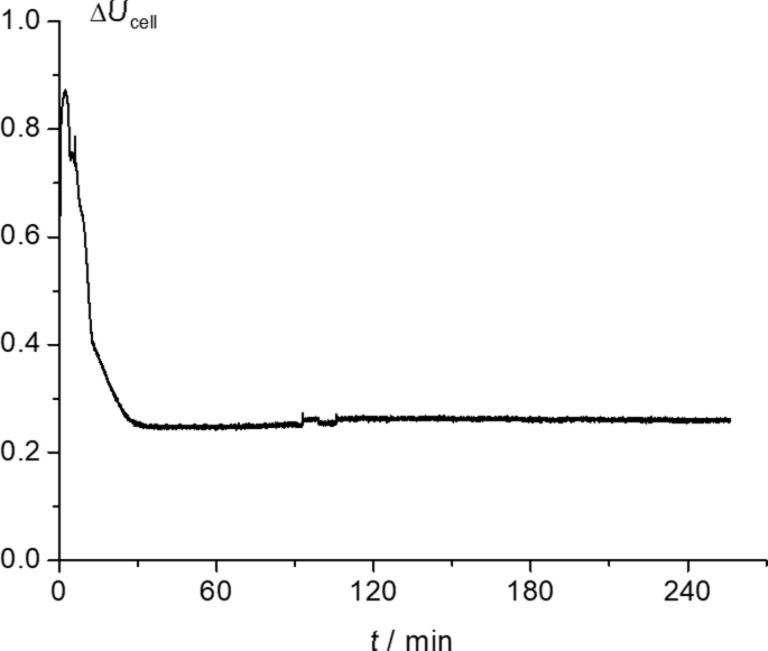
Plot of cell voltage versus time obtained from a preparative electrosynthesis performed at 10 mA cm^−2^, Pd_0.02_/C/T electrode; 0.5 M benzophenone as starting solution in ethanol/water (90:10 v/v) plus 0.1 M H_2_SO_4_.

Table 1 also depicts the average cell voltage from the electrocatalytic hydrogenation of benzophenone, with values of 0.2, 0.3 and 0.5 V for 10, 15 and 20 mA cm^−2^, respectively. More precisely, Figure 6 plots cell voltage versus time at 10 mA cm^−2^. In this case, a high cell voltage increase is observed from the open circuit voltage of around 0 V until the first stage of the electrocatalytic hydrogenation, followed by a cell voltage drop attributed to a decrease in IR drop of the membrane provoked by humidity lack of the membrane at either the anodic or cathodic sides when starting the electrocatalytic hydrogenation reaction. A similar variation of cell voltage versus time was also observed when using the rest of current densities.

**Figure 7:**
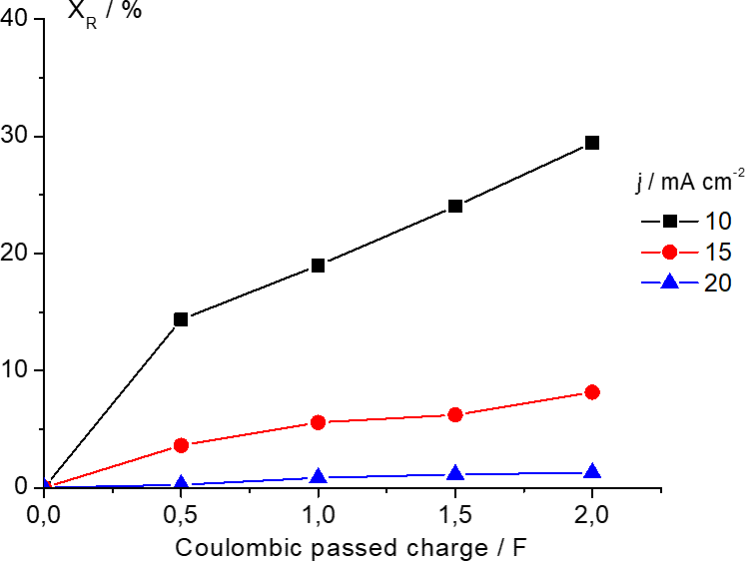
Fractional conversion of benzophenone as a function of coulombic charge passed; 0.5 M benzophenone as starting solution in ethanol/H_2_O (90:10 v/v) plus 0.1 M H_2_SO_4_. Pd_0.20_/C/T electrode.

**Figure 8:**
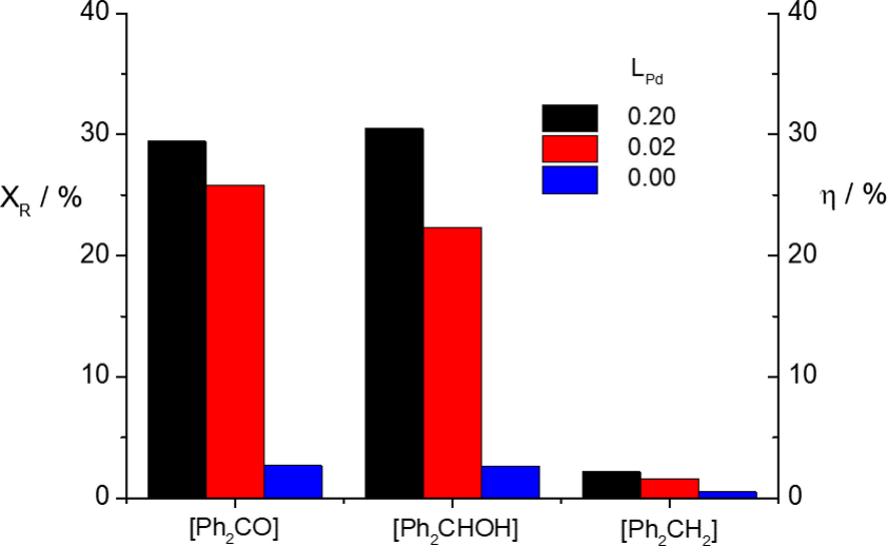
Comparison of fractional conversion (X_R_) and product yield (η) between Pd_0.02_/C/T, Pd_0.20_/C/T and Pd_0.00_/C/T electrodes.

Next we turn out to explore the influence of the Pd electrocatalytic loading on benzophenone conversion. Once again, electrocatalytic hydrogenation experiments were performed at constant current densities of 10, 15 and 20 mA cm^−2^ using a manufactured cathode with a Pd electrocatalytic loading of 0.2 mg cm^−2^ (Pd_0.20_/C/T electrode). Results depicted in Figure 7 show again a higher benzophenone fractional conversion at lower current densities, as happened for the electrocatalytic hydrogenation using an electrode (Pd_0.02_/C/T) with a 10 times lower Pd loading. Interestingly, benzophenone conversion performed at 15 and 20 mA cm^−2^ turned out to be slightly lower for the Pd_0.20_/C/T electrode (vide infra). In this regard, Figure 8 shows the comparative results in terms of fractional conversion and product yield for both electrocatalytic hydrogenation reaction performed at Pd_0.02_/C/T and Pd_0.20_/C/T electrodes and no significantly electrocatalytic hydrogenation reaction at Pd_0.00_/C/T electrode appears. In addition, the selectivity of diphenylmethanol is still over 90% irrespectively of the current density examined; the alcohol and alkane derivatives are also obtained, though prevailing the diphenylmethanol with high product yield (see Figure 8), being similar behaviour as that shown for the electrochemical hydrogenation using the Pd_0.02_/C/T electrode.

The comparison of fractional conversion and product yield displayed in Figure 8 shows that the electrocatalytic hydrogenation of benzophenone is nearly irrespective of the Pd electrocatalytic loading. Indeed, one would respect a much higher fractional conversion of benzophenone when using the Pd_0.20_/C/T electrode. In other words, the higher Pd electrocatalytic loading, the higher adsorption of the organic molecule, and likely the higher reactivity of the adsorbed organic molecule with adsorbed hydrogen. At this point, we have defined a parameter, X_N_, as fractional conversion normalised towards the electrochemically Pd accessible surface area, i.e., Pd accessible sites, according to Equation 3 shown below in order to shed light on the real effect of the Pd loading on the electrochemical hydrogenation of benzophenone.

[3]
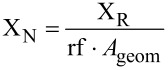


This parameter denotes the usefulness of electrocatalytic loading and is expressed as % (cm^2^ Pd)^−1^ where, X_R_ and rf have the usual meanings and A_geom_ corresponds to geometric area.

We have calculated the X_N_ value accordingly to the optimum current density of 10 mA cm^−2^ for both electrocatalytic loadings. These values are 0.073 and 0.014 for 0.02 and 0.2 (mg Pd) cm^−2^ respectively, confirming higher electrocatalytic usefulness for lower catalytic loading. By comparing Pd electrocatalytic loadings between 0.02 and 0.2, only 16% of the total accessible surface area in Pd electrocatalytic loading of 0.2 mg cm^−2^ resulted to be active to electrocatalytic hydrogenation of benzophenone. Accessibility problems of benzophenone molecules reaching electroactive sites within a thicker electrocatalytic layer are the most plausible explanation for getting a lower X_N_ value. Consequently, an increment of the electrocatalytic layer does not guarantee an enhancement of fractional conversion. For this reason, lower electrocatalytic loadings are the best approach to take into consideration.

**Figure 9:**
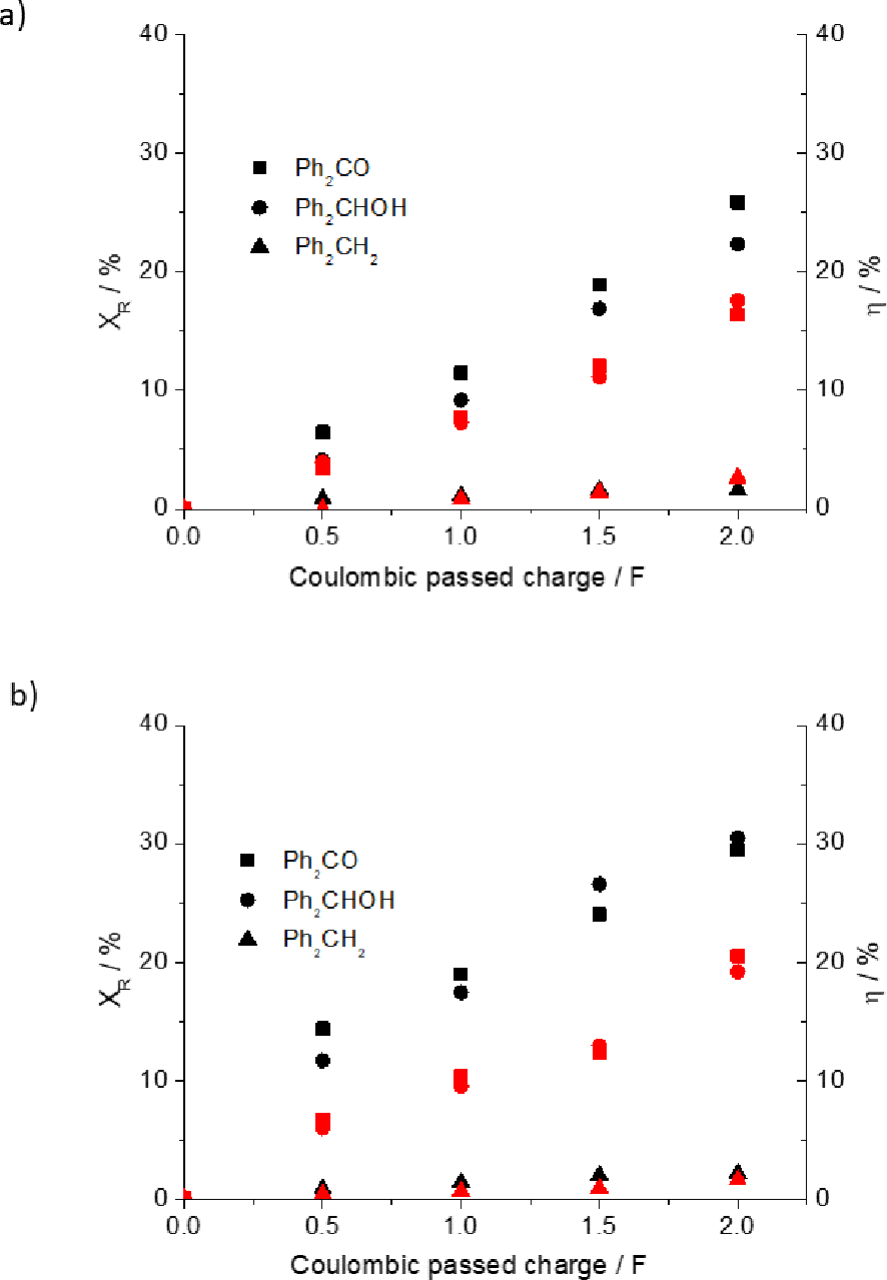
Fractional conversions of benzophenone and product yield of diphenylmethanol at both electrodes. (a) Pd_0.02_/C/T electrode; (b) Pd_0.20_/C/T electrode; 0.5 M benzophenone as starting solution in ethanol/water (90:10 v/v) in the presence of 0.1 M H_2_SO_4_ (black symbols) and absence of H_2_SO_4_ (red symbols).

Finally, the influence of acidic medium in catholyte solutions was explored to simplify the work-up of the crude mixture from the electrocatalytic hydrogenation of benzophenone. It is worth noticing that acidic medium is a parameter that must be taken into consideration in terms of selectivity for electrocatalytic hydrogenations of several organic compounds. In this regard, we performed the electrochemical hydrogenation of benzophenone in the absence of 0.1 M H_2_SO_4_ in order to investigate the contribution of hydronium ion formation from hydrogen oxidation reaction passing through the polymer electrolyte membrane as the sole hydronium ions source reaching the catholyte compartment. In doing so, the electrocatalytic hydrogenation of benzophenone was performed at 10 mA cm^−2^ using both Pd_0.02_/C/T and Pd_0.20_/C/T electrodes in the absence of sulfuric acid. Figure 9 depicts the fractional conversions and product yields as a function of the coulombic charge passed for both electrodes in the absence and presence of 0.1 M H_2_SO_4_ for comparative purposes. Even though no changes were observed in terms of selectivity in the absence of the electrolyte, a remarkable fractional conversion decrease of around 10% can be calculated. As summary, the sole contribution of hydronium ions originating from the hydrogen oxidation reaction is not enough yet for an optimal performance of the electrocatalytic hydrogenation of benzophenone.

## Conclusion

The electrocatalytic hydrogenation of benzophenone has been performed at pre-pilot scale using a polymer electrolyte membrane electrochemical reactor (PEMER). The benzophenone conversion and diphenylmethanol and diphenylmethane yields are driven mostly by the current density used. Even though the lowest current density favours the benzophenone conversion and the alcohol yield, higher current density leads to a high selectivity. In terms of Pd loading within the electrocatalytic layer, no significant difference is achieved with respect to fractional conversion and product yields, however, Pd accessible surface area per weight unit turns out to be enhanced for the lowest electrocatalytic loading, indicating a benzophenone accessibility to the Pd nanoparticulate electrocatalysts.

Hydrogen gas evolution reaction in the cathode compartment can be reused to feed a gas diffusion electrode for the hydrogen oxidation reaction. Moreover, in the absence of an acid medium as supporting electrolyte commonly used at the cathode compartment, the electrocatalytic hydrogenation is feasible by the sole supply of hydronium ions generated at the anode compartment coming from the hydrogen oxidation reaction.

Finally, it is worth noting that the hydrogen oxidation reaction was used as anodic process decreasing cell voltage of the process; besides, the use of hydrogen evolved at the cathode as competitive reaction must be considered as hydrogen source to partially feed the hydrogen anode.

## Experimental

Benzophenone, diphenylmethanol, diphenylmethane, ethanol 96% (Sigma-Aldrich, Spain) were used without further purification. K_2_PdCl_4_ salt was purchased from Sigma-Aldrich (purity higher than 99%). Vulcan XC72R carbon material was purchased from Cabot Corporation. The other chemicals were purchased in the highest purity available and used as received. The solutions were prepared with doubly distilled water.

The synthesis of Pd nanoparticles was performed following a procedure previously used in our laboratory and described in the literature [27–28]. Briefly, a K_2_PdCl_4_ salt solution was reduced using NaBH_4_ as reducing agent in a water-in-oil (w/o) microemulsion in the presence of polyethylene glycol hexadecyl ether, Brij@30 as capping agent and *n*-heptane as organic solvent (water/Brij@30/*n*-heptane). After precipitation and copiously washing with acetone and water, Pd nanoparticles were supported on Vulcan XC72R to get a nominal 30 wt % Pd loading (Pd/C electrocatalyst).

The cathode layer was prepared by air-brushing an electrocatalytic ink onto a Toray Paper TGPH-90 (carbonaceous composite paper supplied by Toray Industries Inc., thickness 280 µm). The electrocatalytic ink consisted of Pd nanoparticles supported in Vulcan XC72R 30 wt % dispersion in isopropanol containing a Nafion dispersion of 5 wt %. All electrodes have a Pd/C to Nafion ratio of 60:40 in the electrocatalytic layer with Pd loadings of 0.20 and 0.02 mg cm^−2^. Cathodes were named as Pd_x_/C/T where x stands for the Pd loading and T is the Toray paper support.

Morphology, size and dispersion of Pd nanoparticles in Pd/C were analysed by a transmission electron microscopy (TEM) using a JEOL JEM-2012 instrument with an accelerating voltage of 300 kV. The TEM was connected with an energy dispersion X-ray (EDX) for the analysis of the Pd nanoparticles. Thermogravimetric analysis (TGA) was performed using a Mettler Toledo model TGA/SDTA851 and /SF/1100 using a heating slope of 10 K min^−1^ from 298 K to 1023 K under a N_2_:O_2_ (4:1) atmosphere. For the characterisation of the Pd_x_/C/T scanning electron microscopy (SEM) micrographs were obtained using a Hitachi S-3000N microscope with backscattered electron signal. Electrochemical surface characterisation of the distinct Pdx/C/T electrodes was explored by cyclic voltammetry (CV). CV measurements were performed using a PGSTAT30 Autolab system. A 0.5 cm × 1.0 cm Pd_x_/C/T electrode acted as a working electrode (WE), the counter electrode was a platinum wire and an Ag/AgCl (3.0 M KCl) acted as reference electrode through a Luggin capillary. CV measurements were performed at room temperature and under argon atmosphere. CV curves were recorded between 0.1 and 1.2 V vs Ag/AgCl (3.0 M KCl) with a scan rate of 50 mV s^−1^ using a 0.5 M H_2_SO_4_ aqueous solution. The electrochemical accessible surface area (ESA) was defined as the Pd electrochemically active surface divided into the total Pd weight according to the total Pd loading, expressed in (cm^2^ Pd) (mg Pd)^−1^, while the roughness factor was defined as the Pd electrochemically active surface divided by the geometric total area, expressed in (cm^2^ Pd) cm^−2^ (vide supra).

**Figure 10:**
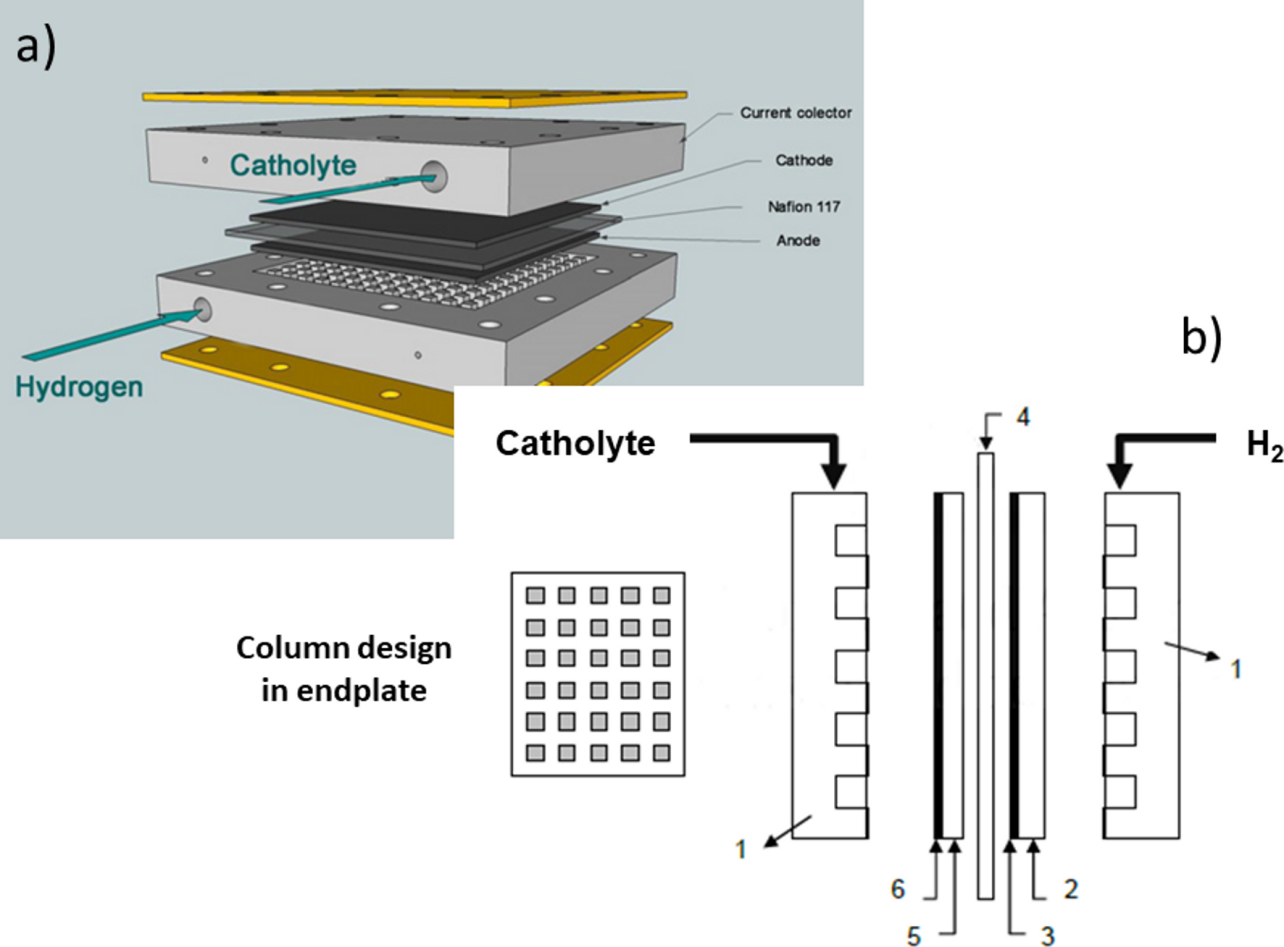
(a) General scheme of a PEMER; (b) itemisation of the main parts of PEMER: 1) endplates, 2) gas diffusion electrode, 3) Pt electrocatalytic layer, 4) polymer electrolyte membrane (solid polymer electrolyte), 5) carbonaceous support (Toray paper TGPH-90), 6) Pd/C electrocatalytic layer.

Electrosynthesis reactions were performed using a 25 cm^2^ PEM single cell fuel cell (EFC-25-01 model from ElectroChem Inc.) acting as polymer electrolyte membrane electrochemical reactor (PEMER), as depicted in Figure 10. Such an electrochemical reactor consisted of (i) a Pd_x_/C/T cathode, (ii) an ETEK gas diffusion-type EFGC (Pt/C/T) with a 40 wt % of Pt with respect to the amount of carbon, and a Pt electrocatalytic loading of 2.0 mg cm^−2^ as anode to carry out the hydrogen oxidation reaction, and finally, (iii) a Nafion 117 cation exchange membrane in contact between both electrodes acting as solid polymer electrolyte.

The catholyte solutions for the electrochemical hydrogenation of benzophenone consisted of either (i) 0.5 M benzophenone in ethanol 96% /water (90:10 v/v) and 0.1 M H_2_SO_4_, or (ii) 0.5 M benzophenone in ethanol 96% /water (90:10 v/v) in the absence of the sulfuric acid electrolyte in order to explore the influence of the medium acidity upon the electrochemical hydrogenation.

A peristaltic pump Ismatel Reglo DIG MS/CA 2–8 provided a catholyte flow of 12 mL min^−1^ through the cathodic compartment and the total catholyte volume was 40 mL. At the same time, hydrogen (Praxair, purity: 99.999%) was fed to the anodic compartment at a flow rate of 50 mL min^−1^ as reactant in hydrogen oxidation reaction, using a MTS-module A-150 from ElectroChem Inc. Electrocatalytic hydrogenation reactions were carried out at constant current densities of 10, 15 and 20 mA cm^−2^ considering a projected geometric area of 25 cm^2^ for both Pd_0.02_/C/T and Pd_0.20_/C/T electrodes using a potentiostat OrigaFlex OGF05A. Total coulombic charge passed was set at 2 Faradays for each experiment. The cell voltage was recorded during each experiment. All preparative electrochemical hydrogenations were carried out at room temperature. In this case, counter electrode plug was connected to reference electrode one and chronopotentiometries were performed registering cell voltage versus time.

The fractional conversion of benzopheneone and product yield and selectivity of final products from the electrocatalytic hydrogenation of benzophenone was examined as a function of current density and Pd loading in the electrocatalytic layer. On doing so, the catholyte solution was analysed by using a high-resolution liquid chromatograph (HPLC Agilent 1200) with a Hypersil ODS column 4 × 250 mm, 5 µm particle size (Agilent Technologies). An acetonitrile/water (1:1 v/v, acetonitrile isocratic HPLC grade and water from Elix 3 Millipore system) was used as mobile phase. The flow rate was 1 mL min^−1^; the injection volume was 100 µL and the working temperature was 303 K. Such experimental conditions provided linear calibration curves between 20 and 100 ppm with all benzophenone, diphenylmethanol and diphenylmethane standard organic solutions. Preparative electrosyntheses were given as fractional conversion (X_R_) of benzophenone (Ph_2_CO) and product yield (η) of diphenylmethanol (Ph_2_CHOH) and diphenylmethane (Ph_2_CH_2_). Selectivity values (ξ) of diphenylmethanol and diphenylmethane were also calculated. For comparative purposes, a free-Pd electrocatalytic layer (Pd_0.00_/C/T) cathode was also considered for the electrocatalytic hydrogenation of Ph_2_CO.
